# Advances in computational frameworks in the fight against TB: The way forward

**DOI:** 10.3389/fphar.2023.1152915

**Published:** 2023-04-03

**Authors:** Akshayata Naidu, Smruti Sudha Nayak, Sajitha Lulu S, Vino Sundararajan

**Affiliations:** Department of Biotechnology, School of Bio Sciences and Technology, VIT University, Vellore, India

**Keywords:** tuberculosis, diagnostic tools, machine learning and AI, drug discovery, insilico analysis, infectious disease management, reverse vaccinolgy, antimicrobial resistance (AMR)

## Abstract

Around 1.6 million people lost their life to Tuberculosis in 2021 according to WHO estimates. Although an intensive treatment plan exists against the causal agent, *Mycobacterium Tuberculosis*, evolution of multi-drug resistant strains of the pathogen puts a large number of global populations at risk. Vaccine which can induce long-term protection is still in the making with many candidates currently in different phases of clinical trials. The COVID-19 pandemic has further aggravated the adversities by affecting early TB diagnosis and treatment. Yet, WHO remains adamant on its “End TB” strategy and aims to substantially reduce TB incidence and deaths by the year 2035. Such an ambitious goal would require a multi-sectoral approach which would greatly benefit from the latest computational advancements. To highlight the progress of these tools against TB, through this review, we summarize recent studies which have used advanced computational tools and algorithms for—early TB diagnosis, anti-mycobacterium drug discovery and in the designing of the next-generation of TB vaccines. At the end, we give an insight on other computational tools and Machine Learning approaches which have successfully been applied in biomedical research and discuss their prospects and applications against TB.

## Introduction

According to the recent report from WHO, around 10.6 million new cases of Tuberculosis (TB) are estimated to be reported in 2021 with around 1.6 million deaths globally (Global Tuberculosis Report, 2022). When left untreated, TB can be fatal and has a staggering 50% mortality rate over the course of 5 years. Major risk factors for disease include - undernourishment, HIV infection, alcohol, smoking and diabetes (Global Tuberculosis Report, 2022). Additionally, a hidden prevalence of latent TB, where the infection persists without clinical manifestations, further pose challenge in HIV positive subjects and in children below the age of 5 with the conversion rate to active TB being around 10% (Latent Tuberculosis Infection Updated and Consolidated Guidelines for Programmatic Management, 2018.)(Mehtani et al., 2021).

*Mycobacterium tuberculosis,* the causal agent of TB infects the alveolar macrophage in the lung tissues which leads to the recruitment of innate immune cells followed by the immigration of adaptive immune cells. As a response, the pathogen employs several virulent factors to attack the local host cells and evade immunological defense mechanisms (Hingley-Wilson et al., 2003) (Pajuelo et al., 2021). Due to the complex and shrewd strategies the bacterium deploys, regular protective responses fail to take off and instead a caseating granuloma (with necrotic center) is formed. Moreover, the pathogen fights against the immune system at different stages of infection. Upon acquiring the bacterium during bacterium invasion, the macrophages become necrotic and secrets interferon alpha/beta and TNF resulting in local lung tissue inflammation. The necrotic state is further maintained by dysregulated neutrophils whose protective actions are inhibited by the pathogen. Some infected macrophages turn into foamy macrophages with altered functionalities providing habitation to the pathogen. Furthermore, *Mycobacterium tuberculosis* actively evades adaptive immune responses by inhibiting neutrophil apoptosis, restricting antigen presentation in the dendritic cells and by encouraging antigen export from the dendritic cells along with maintaining an immune suppressive environment at the site of infection (granuloma) (Pattanaik et al., 2022). In the T cells, glycolipids secreted by the pathogen inhibits TCR signaling during antigen presentation (Chandra et al., 2022).

In 1990s, DOTS (Directly Observed Therapy Short Course) was adopted and introduced by the WHO in the fight against TB. The regime consists of 6 months of sequential treatment with four different antibiotics (isoniazid, rifampicin, ethambutol and pyrazinamide). Although the program was very effective at the beginning, with time, we observed increasing evolution of Multi-Drug Resistant (MDR) *M. tuberculosis (*causal agent*)* at different locations all around the world. Based on WHO report from 2016, out of the 10.4 million cases of TB observed globally in 2015, around 5% were because of MDR strains. It was an alarming observation considering the existing treatment options give limited success against MDR and XDR -resistant to second line of treatment as well and about 11% of MDR TB [(Perwitasari et al., 2022) TB. The success rate of the current treatment regime against MDR TB is around 54% (based on a meta-analysis of 91,538 MDR-TB infected patients (Ahuja et al., 2012)] and a low of 28% for the XDR TB as compared to a triumphant 83% against drug susceptible strains. This presents a grim situation and emphasizes the need for new diagnostic and treatment tools, to identify infected patients and treat them in a timely manner (Khan et al., 2022; Sankar et al., 2022). Moreover, as we stand against a pathogen which learns fast and evolves rapidly to develop resistance against antibiotics, a multisectorial approach with equal emphasis on new drug discovery and on prevention (to limit the spread of the pathogen in the population) of the disease is absolutely essential (Gygli et al., 2017).

In highly endemic settings, WHO recommends administration of the BCG vaccine against TB and it has been in the immunization program for many countries since 1970s for neonates. The effectiveness of the TB vaccine varies greatly based on the population under consideration. Unfortunately, it is known that the induced protection fades with age. Hence, a quest for better vaccines using different platforms have been catching speed in the last 2 decades—(a) Viral vector vaccines (Ad5 Ag58A, ChAdOx1 85A- MVA85A, TB/FLU-04L (mucosal)), (b) Subunit vaccines (AEC/BC02, H56:IC31, ID93 + GLA-SE, M72/AS01_E_), (c) Whole cell vaccine (RUTI^®^, DAR-901 booster, Immuvac, Vaccae™), (d) Attenuated live vector (MTBVAC, VPM1002). The mentioned vaccines are at different stages of pre-clinical and clinical trials. However, certain schallenges remain - primary among them is the complexity of *Mycobacterium tuberculosis* pathogen and lack of our ability to screen out potent protective antigens. Secondly, a detailed elucidation of protective immune response against the pathogen is missing, and hence establishment of reliable co-relates of protection remains a major challenge. Last but not the least, given the heterogenicity of host-pathogen interactions in humans and animal models, there have been discrepancies in the immunogenicity when the vaccine candidate testing moves from preclinical to clinical stage making the process incredibly challenging (Li et al., 2020) (Zhu et al., 2018).

Attending to the immediate need for action, the WHO designed holistic “END TB” strategy intends to bring down TB incidence rate by 95% and TB deaths by 95% by the year 2035 as compared to the figures in 2015. The third pillar of the “END TB” strategy focuses on—“Intensified research and innovation” which urges for the research and development of advanced tools that are effective and adoptable to the current healthcare set up (END TB Stretergy, 2016). Unfortunately, the COVID-19 pandemic had a fatal effect on the execution of programs deployed under the END TB strategy. Firstly, TB diagnosis received a severe blow during the pandemic and the number of reported cases fell by 18% (in 2022). This reduction was most evident in—India, Indonesia and Philippines. Because of this decline, we could expect an increase in community transmission as we now have a population which remain undiagnosed and hence without any treatment and out of the radar of the healthcare system, which put a larger population at risk. Moreover, expectedly, WHO reports a decline of 17% in the people receiving treatment against MDR-TB in 2020 compared to 2019 and a significant decline in global spending on services essential for TB control. Grimly, TB associated deaths also increased between 2020 and 2021 to 1.6 million deaths (equivalent to the number reported in 2017). (Global Tuberculosis ReporT, 2022, 2022).

Given the integrated and urgent global focus against the disease, we cannot fail to notice the advent of new computational approaches now at our disposal (Table 1.) for basic and biomedical research and to inspect the potential role they could play in achieving substantial TB reduction. Computational approaches have already been used in multiple avenues—from the simulation of host-pathogen interactions to the drug discovery against the pathogen and can play an essential role in driving the multi-sectorial approach we intend to take against the disease (Bloom et al., 2017). Given that the coming decade would play a major role in determining our dominance over TB (as per the timeline of the “END TB” strategy), this review article provide details on some important computational concepts with their current and potential future applications in the fight against a complex and whimsical pathogen who has plagued the human population for centuries. The review attempts to - 1) provide details on advances of computational approaches in therapeutic and diagnostic biomarker identification, in compound screening, drug-ligand structural interaction and on vaccine development, 2) discuss possible future development in computational approaches against TB for the development of precise and efficient interventions, 3) to motivate and facilitate researchers/clinicians working against TB to tap into powerful computational resources available at their disposal.

**TABLE 1:** List of Software/Tools which are being used for identification of lead compunds against TB.

Sl. No.	Tools/Software	Description/Algorithm	Link
Active site prediction tools			
1	CASTp	Binding sites and active sites of proteins and DNAs	http://sts.bioe.uic.edu/castp/index.html?1ycs
2	BiteNet	Identification protein binding sites	https://github.com/i-Molecule/bitenet
3	P2Rank	Machine learning based binding site prediction	http://siret.ms.mff.cuni.cz/p2rank
4	PrankWeb	Ligand binding site prediction	https://prankweb.cz/
5	PUResNet	Protein and ligand binding site using deep residual neural network	https://github.com/jivankandel/PUResNet
6	PAR-3D	Predict protein active sites	http://sunserver.cdfd.org.in:8080/protease/PAR_3D/index.html
7	ConSurf	Binding site prediction	https://consurf.tau.ac.il/consurf_index.php
8	Pocket-Finder	Active site prediction	http://www.modelling.leeds.ac.uk/pocketfinder/
9	3DLigandSite	Ligand binding site prediction	https://www.wass-michaelislab.org/3dlig/
10	FINDSITE	Ligand binding site	https://cssb.biology.gatech.edu/skolnick/files/FINDSITE/
11	metaPocket	Ligand binding site predictor	https://www.eml.org/
12	SURFNET	Calculate clefts in protein surface	https://www.ebi.ac.uk/thornton-srv/software/SURFNET/
13	LISE	Ligand binding sites	http://lise.ibms.sinica.edu.tw/
14	POOL	Machine learning based functional site prediction	http://www.pool.neu.edu./
15	MetalDetector	To find metal binding site of protein	https://metaldetector.dsi.unifi.it/
**Molecular docking tools**			
1	AutoDock	Lamarkian genetic algorithm	https://autodock.scripps.edu/
2	AutoDock Vina	Genetic algorithm	https://vina.scripps.edu/
3	FlexX	Incremental construction	https://www.biosolveit.de/download/
4	FlexAID	Protein side-chain elasticity and soft scoring function, based on surface complementarity	https://www.biosolveit.de/download/
5	AutoDock Vina	Genetic algorithm	https://github.com/ccsb-scripps/AutoDock-Vina
	Extended		
6	GalaxyPepDock	Based on interaction similarity & energy optimization	https://galaxy.seoklab.org/cgi-bin/submit.cgi?type=PEPDOCK
7	GEMDOCK	Evolutionary method for molecular docking	http://gemdock.life.nctu.edu.tw/bioxgem/
8	LightDock	Protein-protein, protein-DNA, protein-peptide docking	https://lightdock.org/
9	Dockthor	Protein-ligand docking	https://www.dockthor.lncc.br/v2/
10	SwissDock	protein-small molecule interactions	http://www.swissdock.ch/
**QSAR tools**			
1	AZorange	ML based QSAR modelling	https://github.com/AZCompTox/AZOrange
2	CODESSA	Descriptors calculation for QSAR studies	http://www.codessa-pro.com/
3	QSARINS	QSAR modelling	http://www.qsar.it/
4	CORELSEA	For QSAR and QSPR	http://www.insilico.eu/coral/CORALSEA.html
5	McQSAR	Generates QSAR equations using the genetic function approximation paradigm	http://users.abo.fi/mivainio/mcqsar/
6	AutoQSAR	To make high-quality, predictive QSAR models	https://www.schrodinger.com/
7	GUSAR	To create QSAR/QSPR models on the basis of the appropriate training sets	http://www.way2drug.com/gusar/index.html

**FIGURE 1:**
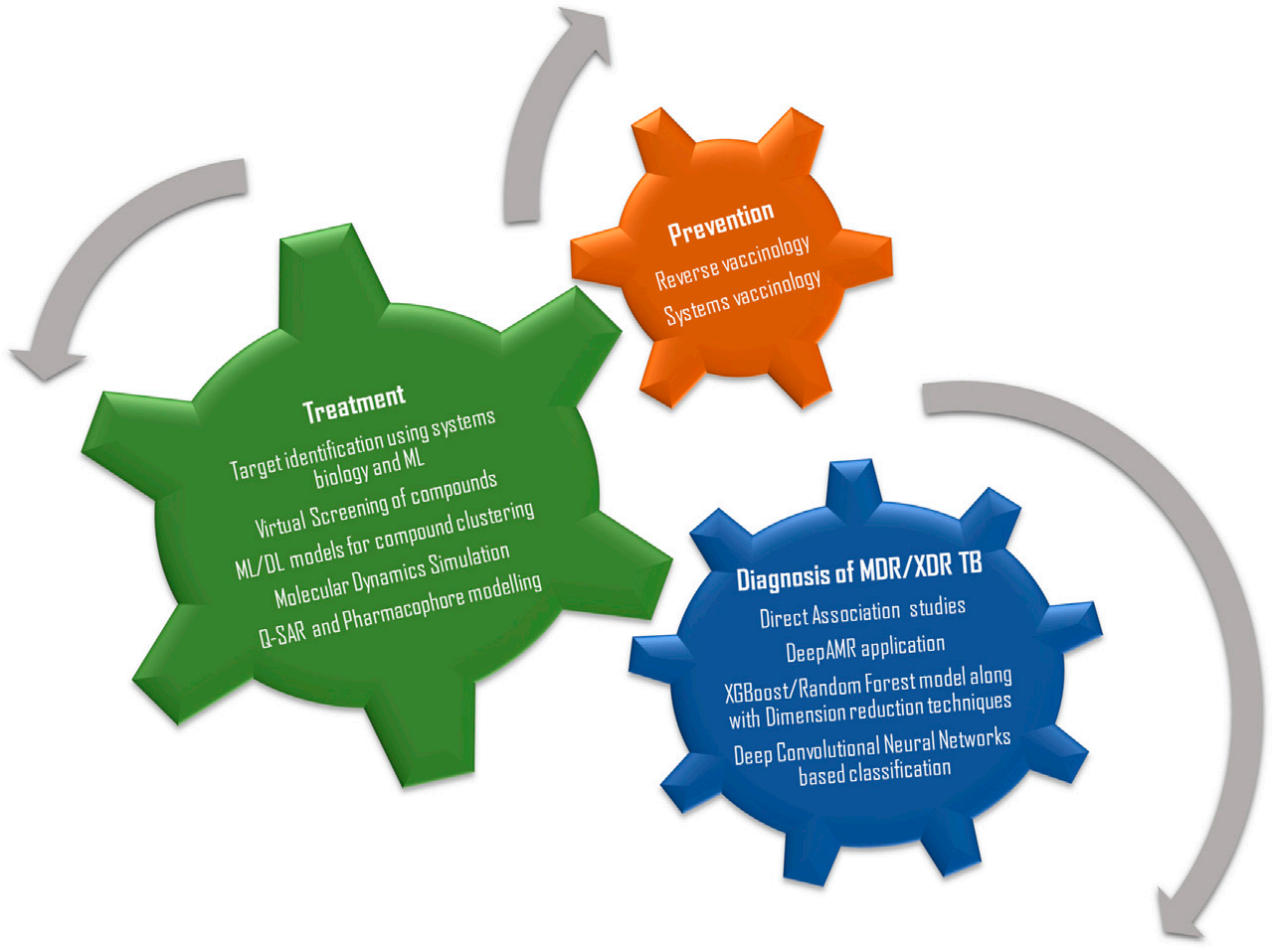
The three wheels of research and development essential for TB management.

For better understanding, the review has been divided into four parts, the first section focusses on applications of computational and systems biology tools in the identification of diagnostic and therapeutic biomarkers against TB, the second section focusses on methods and algorithms used for drug screening and on drug-target interaction studies, the third section briefly discusses on the young and thriving branches of systems and reverse vaccinology and the last section details about some fascinating new advancements in the field of computational biology and their prospective applications against TB (Figure 1).

1) Applications of systems and computational for diagnostic biomarker/therapeutic target prediction in TB

Identification molecular mediators involved in the pathogenesis of a disease or in infection control can have a myriad of applications including—establishment of diagnostic biomarkers, discovery of novel drug targets, identification of biomarkers for monitoring treatment regimens and assignments of correlates of protection (CoP) for vaccines and vaccine candidates. Currently for drug development against *Mycobacterium tuberculosis*, designated prime targets include DNA gyrase (Mdluli and Ma, 2008), Leu tRNA synthetase, ATP synthase and proteins involved in cell wall synthesis like DprE1 and MmpL3 (Williams & Abramovitch, 2023) (Dartois & Rubin, 2022). As for novel targets for drug discovery, KatG, Clp proteases, Menaquinone and KatG have shown great prospects (Bose et al., 2021). One of the promising diagnostic marker against TB have been proposed to be lipoarabinomannan (LAM) antigen (MacLean et al., 2019) (Goletti et al., 2016; Wallis et al., 2013).

### Systems biology in biomarker/target identification

Tools and principles of systems and computational biology have unleashed fresh prospects in developing understanding of host pathogen interactions and for the identification of novel disease associated biomarkers/targets. The advent of high-throughput technologies has provided us with huge volume of data which have been parallelly followed by increase in our computation power. Moreover, relatively easy acquirement of the retrieved omics data (compared to the past) has opened a new dimension to the thriving branch of systems biology where in results from different scales of molecular biology—including—Proteomics, Transcriptomics, Genomics and Epigenomics can be integrated to understand pathologies and immune responses with higher granularity. As we deal with a complicated pathogen in the current scenario, there is a need for advanced tools to skillfully summarize existing high-throughput data to discover credible biomarkers for different stages of the disease (for diagnosis and monitoring purposes), to single out molecular targets for drug discovery and for the development of advanced vaccines (Chandra et al., 2022). In a classic study, RNAseq data analysis study retrieved 44 host response transcripts unique to TB as compared to other diseases including HLA-DPB1, LHFPL2, HM13, and CD74 (Kaforou et al., 2013).

Network Biology provides logical and extensive opportunities to conduct systemic studies when gene/proteins are designed as nodes and the relation between them are denoted by edges. Other networks that could be constructed and used for data analysis include—gene-protein networks, gene-disease networks and gene-drugs network (Yue & Dutta, 2022). Functional enrichment analysis assigns tentative gene ontologies to the genes under investigation using basic statistical tests like the fisher’s test accompanied by test correction methods like the false discovery rate. Other than this, along with supervised machine learning algorithms, unsupervised machine learning algorithms like principal component analysis and neural networks provide us with an unprecedented scope to understand underlying patterns associated with infections (Eckhardt et al., 2020; Yao et al., 2021). In a sophisticated study, network analysis along with functional enrichment analysis of data obtained from literature successfully helped in identifying genes involved in RTK signalling pathway (BLK, ABL1, and NTRK1) in infected hosts to screen ligands for host-directed-therapies (Korbee et al., 2018).

In what could be considered a similar study from the pathogen’s side, likely co-targets (DNAE1, RecA, Rv0823c) were identified from the network of genes linked with AMR. Co-targets are targets which can be aimed for apart from the regular target given the susceptibility of the later to drug resistance. Similarly, by combining network topological analysis with structural modelling, a group identified acetyltransferases as likely co-targets against MRD and XDR TB. This finding is highly relevant given the functional role of these enzymes in the detoxification of administered drugs (Chung et al., 2013). Another form of networks which deserve a special mention are the metabolic pathway networks. In an investigative study, Mtb specific pathways were retrieved from KEGG database and “chokepoint” enzymes, which enable unique substrate-product reactions in the metabolic pathways, were identified. From the analysis of, 18 chokepoint enzymes were proposed based on their presence in multiple metabolic pathways. These chokepoint enzymes proposed as drug targets were: HisC1/F, FalK, NarG, PanB/C, Mur-A/E/F, CobU/Y/H, TrpB, narH, trpD, ilvD, ispG, metE (Kushwaha & Shakya, 2010).

As a demonstration of another way of metabolic pathway network analysis, constrain-based metabolic essentiality analysis was performed to calculate metabolic fluxes which reflect the essentiality of a particular metabolite in essential biological functions of the pathogen (like cell growth). This constrain-based tool along with chokepoint enzyme retrieval from the genomic data revealed—chor, mycolate, alaala, cexccoa, udcpp, arab-D, cdpdhdecg, 26dap-M, hpmtria, kmycolate as potential drug targets (Kim et al., 2009). Also, metabolic flux analysis of the pathways involved in carbon dioxide fixation have suggested isocitrate lyase as essential enzyme for pathogen survival and hence as a durable target (Chung et al., 2013).

Other systems biology approaches have been reported in depth and at varying scale for their application in understanding Tuberculosis. A classic review summarized how these approaches with different tools can be used to develop deterministic models at the population level using SIR models (systems of ODE equations) to simulate the transmission of the *mycobacterium* in a population, for example, (Side et al., 2017). Down the line, the same approaches can be used to model the cellular and molecular immune response against the bacterium. Furthermore, at the molecular level, inside a cell, omics data can be used to develop both statistical and deterministic models to simulate immune responses to the pathogen. When these data are obtained and studied on a time scale, they can provide valuable information on the progression of the disease and the host-pathogen interplay (Young et al., 2008). For example, in a study, kinetic modelling using ODEs of the TCA metabolic cycle and glyoxylate pathway revealed isocitrate lyase as a target of interest (Chung et al., 2013).

Transcriptomics and gene expression data have been obtained for TB using both from *in vivo* and *in vitro* samples to first better understand host-pathogen interactions and then to detect early molecular markers of the disease (Young et al., 2008). In a sophisticated *in vitro* study, RNASeq data were obtained from macrophages infected with a virulent and an attenuated strain of *Mycobacterium tuberculosis.* Genes differentially expressed were obtained by mapping the obtained RNASeq reads against the genome and by estimating the read counts. Genes with high read counts (FPKM >10*, Fragments Per Kilobase of exon per Million*) were considered to be differentially expressed and were analysed further. Through comparative analysis, they observed a peculiar positive association of *SLC7A2* to infection from the attenuated strain. To note, that the same gene was downregulated in the macrophages infected with the virulent strain. They further validated their computational finding with *in vivo* studies to propose *SLC7A2* to be of interest in Tuberculosis credited to its function in regulating the bacteria virulence in the macrophages (J. Lee et al., 2019). In another study, differential gene expression analysis from peripheral blood samples were able to distinguish between TB infected and healthy controls using Mann-Whitney test. The study found three biomarkers—*DOC9* (low), *EPHA* (low) and *NPC2* (high) while demonstrating how TB progression can be monitored using these biomarkers in patients (infected) with or without treatment (recovering) (de Araujo et al., 2021).

Furthermore, network biology tools have been used for downstream analyses of the omics data and to retrieve meaningful information (Young et al., 2008). In a study, gene expression datasets from human subjects with pulmonary tuberculosis were used to retrieve differentially expressed genes (DEGs) associated with disease. The resulting gene list was subjected to gene ontology studies before conducting clustering analysis. Relevant clusters were obtained to construct a protein-protein interaction (PPI) network to perform network analysis. Through network analysis the authors characterised the hub genes (genes with high degree score or more connectivity and hence deemed to play an important role in the concerned mechanism of state). The authors backed their findings from literature and proposed *CCL20*, *CXCL8*, and *IL6* as three of the seven gene markers having high correlation of the disease from the host side. They proposed these markers as potential diagnostic markers and molecular targets against the disease (Sun et al., 2020). Similar approach has been used to construct sub-networks demonstrating differential gene expression patterns in different stages of pathogen growth using gene expression datasets. The study revealed potential drug targets involved in Mtb growth arrest–dosR, sigD, hrcA and nadR (at early stages), furB, sigC and sigE (at latter stages). The identified targets ssssshave significant implications specially for the objective of impeding persistent TB (Chung et al., 2013).

Apart from the extraction of differently expressed genes between infected and control samples, omics data can also be analysed using weighted correlation network analysis (WGCNA) which deals with pairwise correlation through the analysis of biological networks. An elaborate study traced on co-expressed genes from gene expression data of samples obtained from different geographical locations (endemic and non-endemic settings) which were mapped on a meta-human-protein-protein interaction (PPI) network to obtain “*common core*” modules using the Dijkstra’s algorithm (used to single out the shortest path in a network). The modules underwent functional enrichment analysis to reveal *STAT-1* induced proinflammatory responses as prominent expression pathway in all the datasets selected for the study along with giving insight into modules differing in different perturbations (datasets)(Sambarey et al., 2017). Apart from association studies of gene expression profiles to a particular state or condition, the expression values of the genes could also add great value in the calculation of metabolic flux analyses in constrain-based modelling (Chung et al., 2013).

### Screening of non-coding RNAs as biomarkers

Several non-coding RNA including microRNAs, long non-coding RNAs and circular RNAs have been associated with the pathogenesis and immune modulation by the pathogen and have been proposed as biomarkers for diagnosis (including for drug resistant strains) and as prospective drug targets (Kundu & Basu, 2021; Tamgue et al., 2021; Liang et al., 2022) (Alipoor et al., 2020; Sinigaglia et al., 2020; Ostrik et al., 2021). Omics technologies have also been used in establishing biomarkers of TB infection at different stages of infection and also upon treatment. A review comprehensively enlists different miRNAs that have been characterised using RNASeq data analysis from the blood samples and individual blood cells of TB subjects using retrieval of differentially expressed genes. Upon summarizing the results of around 40 studies, the authors highlight some promising candidates as potential biomarkers which are specific for active TB (and are not upregulated in healthy or latently infected subjects)—MIR-133-3p, MIR-26A-5p and MIR-155-5p (Sinigaglia et al., 2020).

Simple yet effective tools like—positive or negative likelihood ratios have been used to identify microRNAs as reliable diagnostic marker against TB (X. Li et al., 2021). In a study, multivariate logistic model and relevance vector machine models were used to profile microRNAs to different categories of TB infection in adults and in children with the authors suggesting prospects of this approach in assigning miRNA signatures to different population (Miotto et al., 2013). The study found MIR-146a, MIR-30e, MIR-600, MIR-223 and MIR-532-5p to be associated with TB in the children group and MIR-25, MIR-365 and MIR-16 to be associated with the infected adult sample group. In another study, miRNAs, small nucleolar RNA and other categories of non-coding RNAs were used to distinguish TB patients. The study found that logistic regression can effectively distinguish between patients with and without TB while proposing four microRNAs (taken together) as sensitive classifiers in diagnosing TB (de Araujo et al., 2019). Moving towards more advanced and clinically suitable approach of diagnosis, a consortium of research groups developed a model to detect TB patients using circulating microRNAs present in the serum (within the 6 months of the disease). For doing this they used different ML algorithms, namely,—Random Forest, Neural Networks, Support vector machine and Elastic-net Logistic Regression to distinguish between patients with TB and adult household contacts (controls). The authors validated their results using leave-one-donor-out-cross validation (LOOCV). Elastic-net logistic regression was shown to be the best performer with an AUC of 0.7 (Duffy et al., 2018).This study skilfully demonstrated the potential of extracellular circular miRNAs in discriminating subjects with active TB from healthy controls. In another important study, electronic health records were also included along with exosomal RNAs to develop a support vector machine model which worked with a high AUC of 0.97 in distinguishing TB patients from healthy controls (Hu et al., 2019).

An all-encompassing review article lists down the protocols and tools which are available for identifying and profiling non-coding RNAs associated with particular conditions along with listing relevant databases for non-coding RNAs. In the review article, miRWalk 2.0, MatureBayes, Starbase v2.0 and CircRNAbase tools have been listed as sophisticated tools to study interactions with the biomolecules with targets and with each other (Almatroudi, 2022).

### Screening of diagnostic biomarkers using machine learning and deep learning

Machine learning and deep learning models have found their applications in detecting and monitoring TB using clinical and molecular data. Attempts have been made to use CT and radiomics data as input to a deep learning model for TB diagnosis (Nijiati et al., 2022) while genetic data are increasingly being used to detect antibiotic resistance. A combination of these methods can be used to monitor the disease progression in the infected individual and to keep track of the spread of Mtb on the population level (Liang et al., 2022).

Early identification of drug susceptible or resistant variants of Mtb is of outmost importance (Swain et al., 2020) to chart out the treatment options and have been conventionally been relied upon on culture-based detection of susceptibility which is quite time consuming and expensive. The large scale WGS sequencing facilities established globally to detect coronavirus in the wake of the pandemic provide an unprecedented opportunity for accurate diagnosis of AMR TB. Sequencing of the whole genome of this complex microorganism will not only provide information on drug susceptibility but will also highlight the genetic islands of susceptibility and resistance along with indicating other genes of significance involved in disease transmission and severity. Given the prospects, resources are required to be directed towards developing a standardised bioinformatics pipeline for diagnosis (with special emphasis on drug resistance detection) and in creating a safe and easy-to-interpret environment for data sharing, processing and high-end computation (Walker & Crook, 2022). Efforts in this direction have already begun.

Direct Association (DA) studies use WGS data which compare the recently obtained fastq files with pre-exiting list of mutations or databases already associated with resistance to predict if a strain is resistant using classic statistical approaches. Apart from DA, ML algorithms are being looked upon to build robust models to predict drug resistance. A review gives a brief on ten studies which have used ML algorithms to predict AMR. In one study, 23 mycobacterial genes (including eis, gidB, rrs, tlyA, rspL rspA, gyrA, ahpC, fabG1, inhA, katG, rpoB, embB, pnca) and their surrounding base-pairs were used as features to develop machine learning models using different algorithms which were then validated using DA predictions (Walker et al., 2015) (Sharma et al., 2022). Another study retrieved 222 prominent features for resistance prediction using the Multi-task Wide and Deep Neural Network with fastq files obtained from whole genome sequencing which showed high efficacy. This was followed by development of another model—Wide and Deep Neural Network model, with still better accuracy (Green et al., 2022) (Sharma et al., 2022). Other algorithms used for this purpose and which showed high accuracy include Classification Trees and Gradient Boosted Trees, which was used to unravel new mutations which can concur resistance to fourteen drugs. The important feature sets for predictive model building included: Single Nucleotide Polymorphisms (SNPs) observed in genes linked with resistance and SNPs in genes which are con-current with resistance. The authors of the research study highlighted that along with prediction this model could be used to rank different features based on their importance (Sharma et al., 2022a).

Other machine learning algorithms have been used to classify Mtb isolates based on resistance using the presence or absence of specific SNP in genes of interest (Yang et al., 2018). A comprehensive study tested different ML based classifiers and dimension reduction techniques on *Mycobacterium tuberculosis* isolates from different countries and against 11 different drugs. Through the results the authors confirmed the prospects of ML models in predicting resistance against different drug. Upon comparison, gradient tree boosting and logistic regression seem to have better performance than other algorithms. The author pointed out that the said models when accompanied by dimensional reduction (using PCA and non-negative matrix factorization) further enhanced the performance by 10%–15%. The authors also propose the application of these models in identifying new markers of resistance (Kouchaki et al., 2019).

Support Vector machines and Linear Regression methods were also able to rank features and correlate lineage specific mutations with second-line drug resistance. A study used Stacked Ensemble algorithm using structural, physiochemical and evolutionary features to capture resistance against caprepmycin. The author also noted that, features associated with protein sequences were better in predicting resistance than features associated with genomic sequences. Moreover, another study using Multiple XGBoost and Random Forest model concluded that genes of importance were distributed all around the genome. Lastly, it was reported that the performance was enhanced when machine models were used in conjecture with dimensionality reduction techniques like the sparse principal component analysis and non-negative matrix factorization algorithm (Sharma et al., 2022). Moreover, a recent study confirmed dinucleotide frequencies to be an encoding system of interest for representing genomic variations in machine learning algorithms to classify drug resistance in Mtb isolates (Müller et al., 2021).

In a more advanced approach, DeepAMR have been used to detect TB strains resistant to multiple drugs by combining an auto-encoder and layers of classification where each layer would represent resistance to a particular drug—representing a multilayer classification system termed as ensemble classifier chain. The input for the model is a dataset denoting presence or absence of particular SNPs in a given sequence. Based on sensitivity analysis the authors were also able to characterize which feature set (defined be a sets of SNPs) heavily influence the classification and chalk out the genetic correlations of drug resistance. For example, the study found that there is high correlation between resistance to isoniazid and resistance to rifampicin (cross-resistance) (Yang et al., 2019). A 1-D architecture of convolutional neural network was developed to include genetic and non-genetic features to predict antibiotic resistance with accuracy for five different antibiotics. The input matrices (21 × 4) consisted of a 21 base reference window for each SNP of interest. The stated deep ML model slightly outperformed logical regression and random forest algorithms in predicting resistance (Kuang et al., 2022).

But here it is important to note that deep learning models are like dark boxes and the intrinsic know-hows remain unknown even if the predictive efficacy is high. Given this, an innovatory ensemble algorithm adopted by researchers from Boston to investigate genetic causative factors which drive antibiotics resistance in Mtb strains require a special mention. The researchers developed a 1-D deep convolutional neural network to categorise strains as resistant or susceptible with high efficacy. After screening out the most influential genes which affect Deep Convolutional Neural Network (DCNN model performance, mutations in these genes were further characterized using support vector machine algorithm which uses a hyperplane to distinguish between resistant and susceptible strains. The method overall delivered an impressive accuracy of 93% along with highlighting genes (embB, gyrA and pncA) and respective mutations of interest (Zhang et al., 2021).

In another project convolutional neural networks were successfully used to retrieve and characterize known and novel genetic loci linked with single or multi drug resistance (with AUC ranging from 80.1% to 99.5%). The input layer for the model were genomic loci sequences along with regulatory elements around the loci from strains resistant and sensitive to different drugs. The architecture of these networks involved 3 pairs of convolutional layers along with two max pooling layers and the output layer was designed to calculate the probability of resistance to the 13 antibiotics being considered in the study (Green et al., 2022). The DeepLIFT algorithm was used to further characterize the loci of interest (in other words, for feature extraction), in order to pinpoint nucleotide sites of interest in relation to drug resistance. For this purpose, reference genome of a susceptible strain of the bacteria was used. This analysis revealed 18 novel SNPs with their prospective role in resistance—acpM-kasA, gid, rpsA, clpC, embCAB, aftB-ubiA, rss-rrl, ethAR, oxyR-ahpC, tlyA, katF, rspL, rpoBC, fabG1-inhA, eis, gryBA, panD, pncA (Deeplift: Docs, Community, Tutorials, Reviews | Openbase, 2022) (Green et al., 2022).

### Screening of therapeutic targets using machine learning and deep learning

Genomic studies can play an important role in identification of target gene candidates through the process of functional annotation. In a thorough study, transposons insertion sequencing (Tn-Seq) was used to identify genes of interest in Mtb as the isolates were cultured in enriched and minimal media. The objective of the research was the identify universally essential and conditionally essential genes in the metabolic pathways of the pathogen using comparative genomics. This study demonstrates a bioinformatics pipeline which can streamline the identification of conserved genes among dynamically changing Mtb variants and hence can propose strategic target genes (purK, purC, purB, purH) for intervention against disease progression (Minato et al., 2019).

**FIGURE 2:**
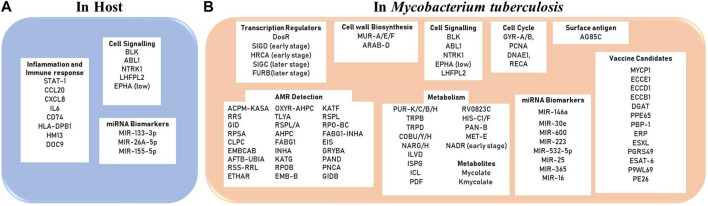
Diagnostic/Therapeutic Biomarkers reported using in silico approaches **(A)** in the host (humans) and **(B)** in the pathogen Mycobacterium tuberculosis.

Extreme Gradient Boosting (XGBoost) is a machine learning algorithm which works as an ensemble of decision trees arranged in a stage-like manner to enhance the predictive capacity of the model. The algorithm has been used before to identify and predict draggable target proteins in humans. The model worked by taking protein sequences as input, where these sequences were encoded using—grouped dipeptide composition (where amino acids are grouped based on their physiochemical properties), reduced amino acid alphabets (where amino acids are represented as clusters based on physiochemical properties and structural similarities), pseudo amino acid composition segmentation (which represent amino acid frequency in a sequence and sequence correlations) (Sikander et al., 2022). An exhaustive implementation of the algorithm in the screening of anti-TB drug targets at different stages of the infection is still pending. Figure 2 summarizes key biomarkers/drug targets identified using *in silico* approaches while Table 2. lists different systems and machine learning algorithms that have been used for biomarker identification against TB.

2) Applications of computational approaches in drug discovery for compound screening against TB

**TABLE 2:** Algorithms and tools used for screening and identification of biomarkers against TB.

Objective	Algorithms/Tools
**Identification of Biomarker using Systems Biology**	• Differential Gene Expression Retrieval (from Microarray and RNASeq dataset)
	• Network Topological Analysis (Hub gene analysis)
	• WGCNA Analysis
	• Metabolic Pathway Networks—Chokepoint Enzyme detection
	• Dynamic Network Analysis (Ordinary Differential Equation (ODEs))
**Screening of non-coding RNA**	• Random Forest
	• Neural Network
	• Support Vector Machine
	• **Elastic-Net Logistical Regression**
**Screening Diagnostic Biomarkers (incl. AMR detection)**	• Direct Association studies
	• Principle Component Analysis
	• Logistic Regression
	• Classification Trees
	• Gradient Boosted Trees + PCA
	• Random Forest
	• **Wide and Deep Neural Network model**
	• Convolutional Neural Network (CNN)
	• Deep Convolutional Neural Network (DCNN)_
	• DeepAMR Algorithm
	• DeepLIFT Algorithm
**Screening of Therapeutic Targets**	• Tn-Seq Analysis
	• WGS Analysis
	• Extreme Gradient Boosting (XGBoost) Algorithm

Currently, several new-generation antibiotics are at different stages of clinical trials and possess varied mechanisms of action. Bedaquiline, the top most drug candidate which has passed through the clinical trials and is under regimen development, attacks the ATP synthase pump of the bacterium. Pretomanid and Delamanid, also in the advanced stages of clinical development conditionally target the electrochemical cycle of the pathogen and intervene in the synthesis of mycolic acid (prominent cell wall component of Mtb species). Promising drugs currently under phase II and III clinical trials include—SQ109, which inhibits MmpL3 and TBA7371, OPC-167832, and BTZ043 which disables DRP epimerase (involved in the biosynthesis of D-arabinose, a component of the Mtb cell wall) (Dartois & Rubin, 2022). Drug discovery for host-targets which could induce immunomodulation are still under early stages of development, i.e., target identification and validation. Along with the above-mentioned drug candidates, given the rise of MDR/XDR, the hidden burden of the disease, and the changing host-pathogen dynamics in the course of infection, a multitude of drug options would be required to achieve TB elimination.

**FIGURE 3:**
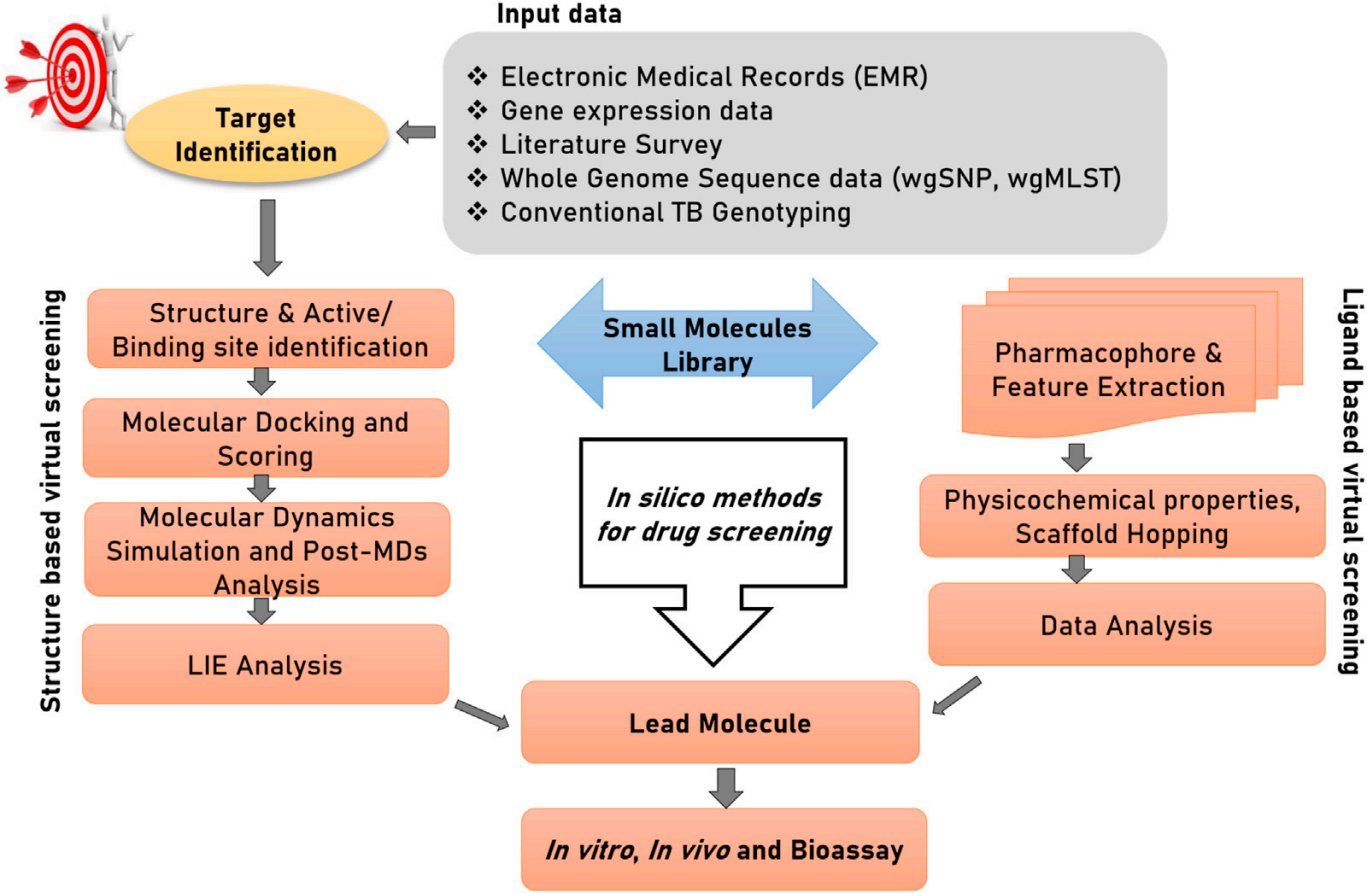
Structure-based and ligand-based drug designing pipeline. The first step of drug discovery is protein target dentification, in which disease-modifying target proteins could be identified through electronic medical records (EMRs), gene expression data, whole genome sequencing data; specifically whole-genome single nucleotide polymorphism (wgSNP) to categorize SNPs that distinguish isolates in a genotype matched cluster, whole-genome multiocus sequence typing (wgMLST), and conventional TB genotyping data which examine specific regions in M. tuberculosis genome and distinguish strains based on gain or loss of segments of DNA sequence. Compound library preparation could be done through various databases and different virtual screening and pharmacophore modelling could be done to select a potential lead compound against target protein. Afterward to confirm their interactions and conformational flexibility, molecular docking and MDs could be performed. Finally, to calculate binding energy of protein with ligand and solvent, linear interaction energy can be done to produce a potent inhibitor.

Computer-aided Drug Design (CADD) or *in silico* drug design can be differentiated into three stages of—1) target identification, (discussed in the above section), 2) compound/ligand screening and 3) structural characterization of the drug-target complex. For the convenience of readers from bio-medical background, we start this section with an introduction of computational tools available for drug discovery. Figure 3 illustrates two important branches of *in silico* methods used for drug discovery. Virtual screening (**VS**) is a procedure to identify leads (drug candidate) across a large and extensive library of bioactive molecules. Traditionally, VS associated techniques have been divided into two major approaches: 1) Ligand-based virtual screening, 2) Structure-based virtual screening. Both of these techniques have been used extensively used for drug discovery against TB. Supplementary material Table 1 provides a detailed description of the basic concepts involved in CADD

***Ligand-based Virtual Screening*** ranks candidate drug molecules based upon their properties and how similar they are to existing active compounds (Motamen & Quinn, 2020). Molecular representations are used for input compounds to screen out a subset of active compounds from a pool of compounds of interest based on existing structure-activity information (Urbina et al., 2021) (Heo et al., 2022). Similarity and internal structure searching, quantitative-structure activity relationship (QSAR) and pharmacophore-based search are examples of ligand-based methods (Jankute et al., 2017).

As we direct our resources towards the fight against TB, it is essential to integrate knowledge generated over 70 years of *in vitro* and *in vivo* experiments conducted for drug identification. With such large volume of data, machine learning algorithms can act as facilitators in retrieving lead compounds with desired structural and functional properties (Mikušová & Ekins, 2017). A research team through a series of publications managed to illustrate the utility of Bayesian algorithm in screening out small compounds with potent anti-tubercular activity (with target selection through whole cell screening) using molecular descriptors (mainly representing structural properties) (Ekins et al., 2013; Ekins et al., 2014) (Ekins et al., 2013). Though it is important to note that, the authors recommended the use of an ensemble (collection of machine learning algorithms) of ML models for better prediction accuracies (Ekins, Freundlich, et al., 2013).

Machine Learning approaches have been proposed to have great prospects in the drug discovery process as its application involve development of classification models based on anti-bacterial properties and extraction of important features which would define these properties. ML algorithms have been used before to screen out small molecules, natural compounds and antibacterial peptides from established databases. Molecular descriptors and fingerprints along with SMILE strings (of the drug candidates) were key input formats for model development and deployment (Jukič & Bren, 2022). Graphical properties, like molecular shape indices and molecular connectivity indices have been used previously to represent physiochemical properties of small compounds. These graph-based signatures have been used to develop a freely available ML application (mycoCSM) to predict inhibitory properties of the compound against Mtb, which when combined with conventional virtual learning approaches can screen out high quality hit compounds (D. E. V. Pires & Ascher, 2020)(Blundell & Pires, 2015) (Jukič & Bren, 2022). In another sophisticated study, ChEMBL-NTD database and the TCAMS (from GSK) database were used to build and evaluate several ML models and these models were evaluated based on precision, sensitivity, specificity and accuracy to reveal AdaBoost decision tree (ABDT), k-Neural Network architecture and Random Forest classifier as the best performers. For model development, the authors considered three sets of molecular descriptors—molecular property descriptors, kappa descriptors and constitutional descriptors. After a screening process through the chi-square test a total of 14 descriptors were included as features in these models (Wani & Roy, 2022).

Given the need of precise intervention because of the advent and progression of AMR, the prospects of anti-tubercular peptides seem very promising - as a therapy against the disease. Given this, authors have contributed to the development of machine learning model - iAtbP-Hyb-EnC which uses an ensemble of ML algorithms (Probabilistic Neural Networks, K-Nearest Neighbour, Fuzzy- K-Nearest Neighbour, Random Forest, Support Vector Machine) to classify peptides based on their activity against M.tb. The protein sequence and physiochemical properties along with one-hot-encoding formed a heterogeneous feature vector and the model delivered an accuracy of 92.68% (Akbar et al., 2021). In another study, XGBoost algorithm showed higher performance than SVM and Random Forest algorithm in the detection of “active” molecules of the pathogen that could be used as a target for intervention (Ye et al., 2021). In another research study, researchers developed a cheminformatics approach to select targets of interest to conduct *in vitro* studies. A Bayesian machine learning model was used to differentiate chemical compounds based on their activity and toxicity in the cellular environment. The highest scoring compounds along with the *in silico* detected target was then taken forward to *in vitro* studies (Ekins et al., 2015). The results of the study suggest BAS 04912643 and BAS 00623753 as promising drug candidates against TB. Table 3 provides detailed list of compounds which have been deduced using *in silico* approaches.

**TABLE 3:** Studies with Quantitative-Structure Activity Relationship (*QSAR)* techniques have been used for TB (LBDD).

S. No.	Method	Target used	Lead molecule	Role in TB	References
1	• 3D-QSAR model	MDR/XDR	(6S) 2-Nitro-6- {[4-(trifluoromethoxy) benzyl] oxy}-6,7-dihydro-5H-imidazo [2,1-b] [1,3] oxazine	Increase the risk of death	Chaudhari & Pahelkar (2019)
2	• QSAR	Pks13	Coumestan 48	It abbreviates fatty acyl chains to produce α-alkyl β-ketoesters	Zhang et al. (2019)
	• ADMET				
3	• 3D-QSAR	GlfT2	5-arylidene-2-thioxo-4-thiazolidinone	catalyzes the elongation of galactan chain	Ortiz et al. (2019)
	• Molecular docking				
4	• QSAR	InhA and PS	Lignans and Neolignans	Activity of dissociative type 2 fatty acid synthase	Maia et al. (2020)
	• Molecular Modeling				
	• ADMET				
5	• QSAR by Austin Model 1 (AM1)	MTB C171Q (KasA)	Xanthone	Fatty acid synthesis	Yuanita et al. (2020)
	• MLR				
6	• QSAR model using MLR	DNA gyrase	Quinoline	ATP hydrolysis	Adeniji et al. (2020)
7	• QSAR	ATP synthase	Quinolines	Respiratory electron flow	Saxena & Alam (2020)
8	• QSAR based on MLR	MtbH37Rv strain	Cinnamic acids	Production of cultural filtrate proteins	Teixeira et al. (2020)
9	• QSAR	DprE1, InhA, PS, and DHFR	Compound 11026134 from benzothiazinone derivative	Synthesis of thymidylate	Viana et al. (2020)
	• molecular modelling				
	• pharmacophores				
10	• QSAR	strainStaphylococcus aureus and *Mycobacterium tuberculosis*	(2*E*)-*N*-(4-bromo-3-chlorophenyl)-3-phenylprop-2-enamide	Cause infections in soft tissue	Kos et al. (2020)
11	• QSAR	PKS13	Imidazo oxazines	It abbreviates fatty acyl chains to produce α-alkyl β-ketoesters	(B & M. K, 2020)
	• Docking study				
12	• SAR analysis	H37Rv	Coumarin	Production of cultural filtrate proteins	Pires et al. (2020)
13	• 4D-QSAR model	DHQase II	Shikimate	Catalyzes the shikimate pathway	Miranda et al. (2021)
14	• SAR	Pks13	4H-Chromen-4-one	It abbreviates fatty acyl chains to produce α-alkyl β-ketoesters	Wang et al. (2021)
15	• 3D-QSAR	DNA gyrase	Triazole	ATP hydrolysis	Adeniji (2021)
	• Virtual screening				
16	• 3D-QSAR	Rv2421c	Coumermycin	Growth of *Mtb*	Cloete et al. (2021)
17	• QSAR: regression	dormant MTB	1,2,4-triazole	Non-replicating state of *Mtb*	Aher and Sarkar (2022)
	• classification based				
18	• QSAR	InhA and DprE1	Thiosemicarbazone	Production of lipoarabinomannan and arabinogalactan	Valencia et al. (2022)
	• MDs				
19	• Synthesis	InhA and DprE1	N-(4-phenoxy phenyl)-7H-pyrrolo [2,3-day]pyrimidin-4-amine	Production of lipoarabinomannan and arabinogalactan	Jesumoroti et al. (2022)
	• SAR				
20	• Atom-based and field based-3D-QSAR models	DprE1	ZINC12196803	Production of lipoarabinomannan and arabinogalactan	Mali et al. (2022)
21	• HQSAR	PknB	Quinazoline	Responsible for the growth of pathogens	Hanwarinroj et al. (2022)
	• 3D-QSAR				
22	• QSAR	strains*Staphylococcus aureus* ATCC 29213 and *Enterococcus faecalis*ATCC 29212 and MRSA and VRE	(2*E*)-3-[3-(Trifluoromethyl)phenyl]-*N*-[4-(trifluoromethyl)phenyl]prop-2-enamide **&**	Facultative anaerobic gram-positive bacteria	Strharsky et al. (2022)
	• Docking study		(2*E*)-*N*-(3,5-dichlorophenyl)-3-[3-(trifluoromethyl)phenyl]prop-2-enamide		
23	• SAR	gram-positive bacteria and two mycobacterial strains	(2E)-3-(3,4-Dichlorophenyl)-N-[3-(trifluoromethyl)phenyl]prop-2-enamide	Cell wall formation	Strharsky et al. (2022)
			**&** (2E)-3-(3,4-Dichlorophenyl)-N-[4-(trifluoromethyl)phenyl]prop-2-enamide		
			**&** (2E)-3-(3,4-Dichlorophenyl)-N-[4-(trifluoromethoxy)phenyl]prop-2-enamide		
24	• SAR	Pks13	1-(1-(4-bromophenyl)-5-hydroxy-2-methyl-4-(piperidin-1-ylmethyl)-1H-indol-3-yl)ethan-1-one	It abbreviates fatty acyl chains to produce α-alkyl β-ketoesters	Cai et al. (2022)
25	• QSAR	DNA gyrase	Triazole	ATP hydrolysis	Adeniji et al. (2022)

***Structure-based screening*** presents the most optimal interaction between ligands and a molecular target (drug-target complex). 3D structure of protein molecules is essential in order to predict the interactions *in silico* (Nayak & Sundararajan, 2023). Target-based VS appears to primarily use the docking concept, in which the three-dimensional structure of a target protein is used to bind with the bioactive molecules and score them based on their corresponding binding score (Djaout et al., 2016; Churqui et al., 2018; Macalino et al., 2020; Srinivas et al., 2021). Strategies which include pharmacophore and protein-ligand fingerprinting, could be used in structure-based VS as well (Macalino et al., 2020). Thus, rather than the total number of hits, the excellence of a VS is marked by the exploration of enticing target scaffolds (Djaout et al., 2016).

Several structural biology approaches have been used to develop therapeutic strategies against *M.tuberculosis* (Prabitha et al., 2022). A recent review article discusses in detail about the different targets which have been used for ligand screening against TB along with providing a list of websites and repositories from which novel ligands can be retrieved (Ejalonibu et al., 2021). In silico tools which can be used for target identification like, homology modelling, virtual screening, molecular docking, and molecular dynamics simulation to identify druggable targets and lead natural metabolites to fight against M. tuberculosis have also been reviewed in other informative articles (Okombo and Chibale, 2017; Rudraraju et al., 2022 (Kingdon & Alderwick, 2021; Swain and Hussain, 2022)). In one study, *M. tuberculosis* genes were choosen druggable target to prepare specific compound library from ZINC database, ChEMBL database, and Enamine REAL database for virtual screening. The author of the study suggested that, in case the structure of the target molecule is not available then we can go for homology modelling either using newly mentioned AlphaFold and deep learning (DL) methods or by using computational tools like SWISS-MODEL, I-TASSER, and Phyre2. After preparation of target and small molecule library, receptor based virtual screening and molecular docking study has been carried out using different tools and software such as; AutoDock Vina, AutoDock tool, CDOCKER, FRIGATE, Glide, Gold, LibDock, and FlexX (Ejalonibu et al., 2021). Most of the articles surveryed have targeted proteins like, MurB, MurE, InhA, DHFR, FabG, cyclophilin A, DprE1, PanK, PknB, KasA protein, Isocitrate lyase, RmID, FtsZ, AroQ, Mbtl, EthR, MraY, NarL, PknA, BioA and LDtB. In an advanced study, machine learning based molecular docking method was used, where the authors reported a target of *M. tuberculosis* called ribosomal peptidyl transferase and performed the ML based virtual screening to boost up the screening productivity (Kovalishyn et al., 2018). Then molecular dynamics simulation (MDs) was performed to get the conformational stability and flexibility of complex using different forcefield parameters like; AMBER, GROMOS, and CHARMM. From the MDs trajectory, clustering approach was performed for getting an representative structure (RS) within a cut-off value by users. Then ensemble docking methods were applied to find anti-TB drugs and to minimizing the disadvantages of receptor-ligand docking. Table 4 provides a list of methods which have been used for structured based drug designing against TB.

**TABLE 4:** List of methods for *Structure based drug designing (SBDD)* against TB.

S. No.	Method	Target	Role in TB	References
1	• VS	Pks13	It abbreviates fatty acyl chains to produce α-alkyl β-ketoesters	Çınaroğlu & Timuçin (2019)
	• MD			
2	• Molecular docking	Pks13	It abbreviates fatty acyl chains to produce α-alkyl β-ketoesters	Cruz et al. (2019)
	• MDS			
	• Binding free energy calculation			
3	• Docking	Rv2984	Intricate in the catalytic synthesis of inorganic polyphosphate	Shahbaaz et al. (2019)
	• MDS			
4	• Docking study	MurB and MurE	Cell wall formation	Rani et al. (2019)
	• MDS			
	• Binding free energy calculation			
5	• Target-based drug screening	InhA	**Activity of dissociative type 2 fatty acid synthase**	Wang et al. (2019)
6	• VS	Calprotectin	Innate immune activation	Gheibi et al. (2019)
	• MDS			
	• MM/GBSA calculation			
7	• VS	ICL	Bypasses two decarboxylation of TCA cycle	Lee et al. (2019)
	• MDS			
8	• VS	LipU	Cellular uptake	Kaur et al. (2019)
	• MDS			
9	• VS	GS	Catalyzes glutamates to glutamine	Kumari & Subbarao (2020)
	• Molecular docking			
	• MDS			
	• Binding free energy analysis			
10	• Receptor based pharmacophore e modelling	KasA and InhA	Fatty acid synthesis	Puhl et al. (2020)
	• Shape-based pharmacophore model			
	• Selection of compounds			
	• Docking studies and *in vitro analysis*			
11	• Pharmacophore model generation	*M. tuberculosis (H37Rv strain)*	Production of cultural filtrate proteins	Naz et al. (2021)
	• molecular docking			
	• MDS			
12	• *in silico* VS	DNA gyrase	ATP hydrolysis	Shallangwa & Adeniji (2021)
	• Molecular docking			
13	• Homology modelling	MmaA1	Transformation process	Veeravarapu et al. (2021)
	• VS			
	• ADME analysis			
14	• *In vitro* study	InhA	Activity of dissociative type 2 fatty acid synthase	Ahmad et al. (2022)
	• Docking			
	• DFT calculation			
	• MDS			
15	• Molecular Docking	InhA	Activity of dissociative type 2 fatty acid synthase	Angelova et al. (2022)
	• MDS			
16	• Molecular docking	Pks13	It abbreviates fatty acyl chains to produce α-alkyl β-ketoesters	Taira et al. (2022)
	• MDS			
17	• Pharmacophore modelling	PPARγ	Regulator of TB pathogenesis	Prabitha et al. (2022)
	• Docking			
	• MDS			
18	• VS	*Mt*ICL	Intracellular infection	Duan et al. (2022)
	• Molecular docking			
19	• Structural characterization	Rv1417 and Rv2617c	Forms transient molecular complexes in the cell envelope	Paco-Chipana et al. (2022)
	• Docking			
	• MDS			
20	• VS	PrpR	Regulate the expression of enzyme tangled in methylcitrate pathway	Rajasekhar et al. (2022)
	• Docking			
	• MDS			
	• MM/GBSA			
21	• Docking	PI3K	It abbreviates fatty acyl chains to produce α-alkyl β-ketoesters	Dasmahapatra et al. (2022)
	• Prime-MM/GBSA			
	• PASS algorithm			
	• MDS			
22	• Molecular docking	CYP51A1	Animal version of cytochrome which involves in the conversion of lanosterol	Munia et al. (2022)
	• MDS			
	• Pharmacophore site identification			
	• DFT calculation			
23	• VS	GlgE	It stretches linear α-glucans	Singh et al. (2022)
	• Drug-likeliness properties			
	• MDS			
	• *In vitro* analysis			
24	• Pharmacophore modelling	MtDHQ	Catalyses the shikimate pathway	Souza et al. (2022)
	• VS			
	• Docking			
	• MDS			
25	• Molecular docking	Rv1417 and Rv2617c	Forms transient molecular complexes in the cell envelope	Aguilar-Pineda et al. (2023)
	• DFT			
	• MDS			

MDS, molecular dynamics simulation; VS, virtual screening.

**Pharmacophore modelling** is a technique that is used to identify and determine potential associations among protein-ligand complexes. The formed association should have uniform electrostatic and mechanical properties, which are required to elicit a therapeutic benefit - it is required to maintain ideal biomolecule interconnections with the use of a distinct molecular target structure in order to block its biochemical reaction (Zhang et al., 2018). In one study, the authors have used structure based and shape-based pharmacophore modelling using Discovery Studio software (DS), molecular docking by using libdock in DS and computational tools for the prediction of inhibitors against KasA in TB (Puhl et al., 2020). They have used a pre-existing structure of DG167 for KasA protein. Also 7 compounds were considered applying MST (Kd = 20–224 μM) out of 62 commercially available small molecules identified from different *in silico* processes. Finally, they found the molecules with high score were ZINC23955828 and sildenafil, which were derived through the whole cell Mtb machine learning model and through the shape-based pharmacophore method respectively. Other molecules which presented good binding score to KasA include chlorpropamide and flubendazole from receptor-based pharmacophore method; lovastatin from shape-based approach; ZINC47871032 and ZINC89983431 from ML models. But, from these compounds chlorpropamide, flubendazole and ZINC47871032 were found to have no significant changes, hence it was concluded that although these molecules could interact with the active site, with no major changes in the conformation of complexations.

**Fragment based drug discovery (FBDD)** involves identification of small molecules as inhibitors to target biomolecules of significance in diseases followed by crystallographic studies to characterise the target-ligand binding (Murray & Rees, 2009). Multiple targets have been identified from the pathogen’s side for the screening of inhibitory fragments including—ArgB (involved in arginine biosynthetic pathway), BioA (involved in biosynthetic pathway), Ag85C and KasA (involved in mycolic acid synthesis), EthR (regulator of mycolic acid production), and DprE1 (involved in the synthesis of arabinogalactan scaffold). More than one hit compounds have been identified to be potential inhibitors of each of the targets mentioned above. For example, Thiolactomycin and Pantetheine analogs have been identified to bind with KasA with promising binding affinities. DprE1 inhibitors as derived through FBDD are at the early stages of clinical evaluation (Mallakuntla et al., 2022; Togre et al., 2022).

The complex pathology of Mtb which involves multiple molecular agents (involved in pathogen entry and sustenance) and the failure of multiple innate immune mediators highlight the need for the adoption of a multi-target approach. Network biology (including topological analyses) approaches have been proposed to be highly beneficial in identifying host or pathogen targets. To identify conformational similarities between the targets identified for the study, a pocket similarity score can be calculated using programs like the Apoc program. After identifying and characterizing drug targets, lead screening can be performed for single or multiple ligands (Srivastava et al., 2019). Around five inhibitory fragments were identified against six known targets with varying function (GyrA, GyrB, InhA, Ag85C, PS, PDF) of the pathogen using QSAR methodologies and the study brilliantly demonstrates the possibility of a multi-target approach against the pathogen (Speck-Planche et al., 2012).

Several compounds have been identified during the last few years with potential anti-tubular properties and are currently undergoing preclinical and clinical studies like, bedaquiline (Antoci et al., 2021; Chakraborti et al., 2021; Patil & Jain, 2021; Deshkar & Shirure, 2022), PA-824 (Lenaerts et al., 2005; Edwards & Field, 2022; Xu et al., 2022; Yan et al., 2022), and delamanid (Skripconoka et al., 2013; Liu et al., 2018; Nasiri et al., 2022). Existing drugs have also been modified to repurpose them against TB and they include -riminophenazines (Valinetz et al., 2020; Brunaugh et al., 2022), b-lactams (Moon et al., 2018; Story-Roller & Lamichhane, 2018; Ur Rahman et al., 2018), and oxazolidinones (Balasubramanian et al., 2014; Alghamdi et al., 2020; Margaryan et al., 2022; Ndukwe et al., 2022). These are examples of how *in silico* approaches have been successfully employed in retrieving promising lead compounds in preclinical and clinical studies. However, so far none of the mentioned compounds fulfilled all the criterion to be labelled as an ideal anti-TB drug. (Cihan-Üstündağ et al., 2019; Appetecchia et al., 2020). Tables 3, 4 provides details about different methods which have been used to identify lead compounds against TB using Ligand-based and Structure-based drug designing approaches respectively.

***Drug Repurposing.*** COVID-19 pandemic has highlighted the need for repurposing validated drugs for use against novel or neglected pathogens. With the rise in drug resistance against Mtb, this approach of drug discovery cannot be more relevant. Dataset with antimalarial drugs were used to screen through target receptors derived from whole cell screening of the *M.tb* using naïve Bayesian algorithm. Such ligand-based screening has also been used against *E. coli* target molecules with several other machine learning algorithms including—Deep Neural Networks, Feed-forward Neural Network and Random Forests (Urbina et al., 2021). Other *in silico* approaches that can be used for drug repurposing include—Phenotypic screening, Target-based methods, Knowledge-based methods, Mechanism-based methods, Signature-based methods, Pathway-based methods and Molecular docking (Kulkarni et al., 2023). Molecular docking has been used recently to study the interaction of 10 FDA approved drugs with two proteins involved in the synthesis of mycolic acid. The study revealed lymecycline as a potent drug (already FDA approved) against Mtb (Umapathy et al., 2021).

***Toxicity Evaluation*.** Machine Learning algorithms have also been used in predicting the toxicity potential of a compound of interest based on its structural similarities to known compounds. These methods are referred as Quantitative Structure- Activity Relationships and Quantitative Structure Property Relationships [Please refer to the Basic Concept section (description of molecular descriptors)]. Apart from it, systems biology approaches are also being used on high through put datasets wherein the effect of ligand-target interactions are evaluated in the host cellular systems based on the molecular chain of reaction this interaction unleashes and their toxicological implications (Vo et al., 2020).

For the development of next-generation of treatment strategy against TB, a multi-dimensional approach of control is only logical given that the bacterium attacks and evades using myriad of inter/intra-cellular molecular pathways. There is a growing support for use of host-directed therapies along with use of anti-microbial drugs in order to train the immune system as a supportive arm while learning to fight against immune-evasion attempted by the pathogen (Chandra et al., 2022).

3) Applications of computational approaches in vaccine development

It is scientifically hypothesized that *Mycobacterium tuberculosis* do not induce a protective immunity in the infected subjects as the chances of re-infection remain high in previously infected patients. Hence, we know preventive vaccines are required to induce a significantly divergent and controlled host responses as compared to the natural immune response. Better characterization of the *mycobacterium* virulence and immune evasion respectively are suggested to pave way for the development of epitope-based vaccines and for prospective vaccines which work on the concept of trained immunity, two approaches a recent review advocate for (Chandra et al., 2022).

The new branch of reverse vaccinology which follows the “genome to vaccine” approach require advanced computational tools to screen through the pan-genome of the pathogen for the identification of ideal vaccine antigens (Vaccinology in Reverse, 2020). Prospective vaccine candidates from several other gram-negative bacteria have been identified through bacterial genome screening and characterization (Pizza et al., 2000; Tettelin et al., 2000). To curate a list of genes from the bacterial genome which would be exposed to the immune mediators upon infection, functional genomics analysis could play an instrumental role and would basically involve clustering and dimension reduction studies in an open-ended manner (to group associated genes/protein according to their functional commonalities)(Bambini & Rappuoli, 2009). Reverse vaccinology has been used before for vaccine design against TB. One of this study revealed six promising novel vaccine candidates against TB which were—PE26, PPE65, PBP-1, Erp, EsxL, PGRS49 (Monterrubio-López et al., 2015)

Molecular docking and simulation studies have also been performed to study the interactions of the designed vaccine with different immunological receptors to check for binding properties and hence resultant potential immune responses. In a sophisticated study, analysis of the bacterial proteome was performed to reveal five proteins of interest for the development of a subunit vaccine. Among the five selected proteins P9WL69 was deemed as a vaccine candidate and was docked against TLR-2, TLR-4, TLR-9, Mannose receptor and MYD88 to characterize the immunogenicity of the selected vaccine candidate. The protein-receptor complexes were further characterized using molecular simulation studies. The study, along with demonstrating the methodology and parameters involved in proteome screening and analysis, also focuses keenly on the potential immunological signalling pathways that can be triggered by the putative vaccine candidate. This excellent study acknowledges the requirement of a strong and divergent innate immune response which is thought to be required to generate durable protection against Mtb (Arega et al., 2021). In similar studies the constructed vaccine assembly with or without adjuvants were docked against TLR-2 (Moodley et al., 2022) and TLR-3 (Bibi et al., 2021) to characterize their immunogenicity further revealing promising vaccine candidates.

In another structural-biology based study, secretory proteins of Mtb were screened for their antigenicity properties to reveal MyCP1, ECCE1, ECCD1, and ECCB1 antigens as best performers. 9-mer and 15-mer peptides were retrieved from these antigens as B-cell and T-cell epitopes and their binding interactions with MHC molecules were characterized with molecular docking and simulation studies to comprehend the potential efficiency of the proposed vaccine candidate to be presented to the lymphocytes. The proposed vaccine candidates were further validated in through *in vitro* experiments (Jagadeb et al., 2021). In another study using pan-proteome screening and epitope prediction suggested two Mtb antigens of importance (DAG acetyltransferase and ESAT-6-like protein) for the development of a multi-epitope vaccine. The interaction of the putative multi-epitope vaccine with both MHC molecules and TLR-4 were characterized using molecular docking and simulation studies (Albutti, 2021). The author of the study in the process of developing a multi-epitope vaccine proposed a consolidated computational framework for the designing of multiepitope vaccine against Mtb.

Apart from reverse vaccinology, the young and thriving branch of systems vaccinology is also expected to play a vital role in out fight against bacterial infections. The field deals with understanding protective immune responses to infection at molecular level and is greatly fueled by the omics data available (especially the gene expression data from host after vaccine administration). Apart from eliciting the molecular chain of interactions unleashed by vaccination, regression studies when employed on the omics data can reveal credible biomarkers or correlates of protection of immune progression which holds high value for immune monitoring and in evaluating vaccine responses. Apart from protection, systems vaccinology along with other computational and ML algorithms can help in delineating molecular mechanisms of adverse events triggered by vaccine candidates in pre-clinical and clinical studies (Pulendran, 2014; Querec & Pulendran, 2014; Fletcher, 2018; Satti & McShane, 2019). Omics data retrieved from vaccinees or from cell cultures can be used to associate gene expression profiles/or SNPs with vaccine induced immune responses using basic statistical tests (like, Wilcoxon signed-rank test to find differentiation of geometric means between the treatment and the control group when dealing with transcriptomics data). Upon BCG administration such a study revealed significant role of hepatic nuclear factor (HNFs) in hematopoietic stem cells and monocytes in inducing—trained immunity (a phenomenon of elevated innate immune system) which have been lately associated with BCG vaccination (Cirovic et al., 2020). A similar genomic study, specific for neutrophil uncovered changes in methylation profile of the leukocyte upon vaccine administration proposing an induced “functional re-programming of neutrophils” as another factor contributing to trained immunity (Moorlag et al., 2020). BCG vaccine has also been reported to have immunomodulatory properties and have been test against several autoimmune diseases like Type-1 diabetes. In a multi-omics association study, expression/demethylation of following genes in the Treg cells were revealed to be associated with the immunomodulatory properties of the vaccine: CD25, CTLA4, CD62L, CD45, TNFRSF18, IKZF2, IKZF4, TNFRSF18, Foxp3, and IL2 (Keefe et al., 2021).

Despite the multitude of studies being conducted to gather the entire picture of the immunological profile associated with BCG, there is a gap of understanding the molecular mechanism through which this vaccine could/would provide protection against TB. Given this transcriptomics/gene expression analysis of the immune responses triggered by Mtb itself can provide important insight into the protective (required) as well as detrimental immune responses (similar to analyses already conducted for other pathogens (Naidu and Lulu S, 2022) which could aid in setting up an target product profile for vaccine development. Efforts in this direction have already begun. For example, gene expression analysis followed by network topological studies have highlighted CTLA4, PRF1, GZMB and GZMA as hub genes associated with latent TB (Zhang et al., 2021). Similar analysis on THP-1 cells, with the use of WGCNA revealed close associated of IL1B, IRAK4 and CCL20 (immune system related genes-among other genes) as being closely associated with MTB infection (Lu et al., 2021).

4) Future prospects

The research community working against TB have made extraordinary progress in employing advanced computational tools to better understand the pathogenesis of the disease and to develop interventions. Yet, given the hefty challenges involved in TB management, more exploration is required to fully utilize and optimize systems and computational tools available at our disposal—for biomarker identification, drug discovery and to find an efficient prevention strategy. In a ground-breaking study, host-pathogen interaction in macaques affected with TB was thoroughly analysed in a temporal manner using imaging, single cell-RNA-Seq and pathogen-clearance-parameter data. The most important findings of the study was that– 1) the timing of intervention (based on the stage of the granuloma) can greatly affect its efficacy of the treatment, 2) TH2 type immune response is negatively associated with infection control and positively associated with high-burden granuloma formation, 3) TH1/TH17 based adaptive immune responses lead to low-burden granuloma formation. Based on these findings, the authors of the study proposed distinct drug targets and hence perspective drugs candidates for different stages of the granuloma formation. Such multi-scale data integration and analysis was conducted using clustering, co-expression, classification and network biology-based algorithms (Gideon et al., 2022.). Along with underlining the indispensability of computational and systems biology tools in the current pro-data research atmosphere, the study signals towards an upcoming glorious period in TB research in humans. Depending on data availability, investigators would be able to comprehend the whole picture of the dynamics of host-pathogen interaction and hence, would be able to come up with precise preventive/therapeutic interventions to regulate/eliminate the pathogen, which is the basis of the thriving branch of Precision Medicine.

**FIGURE 4:**
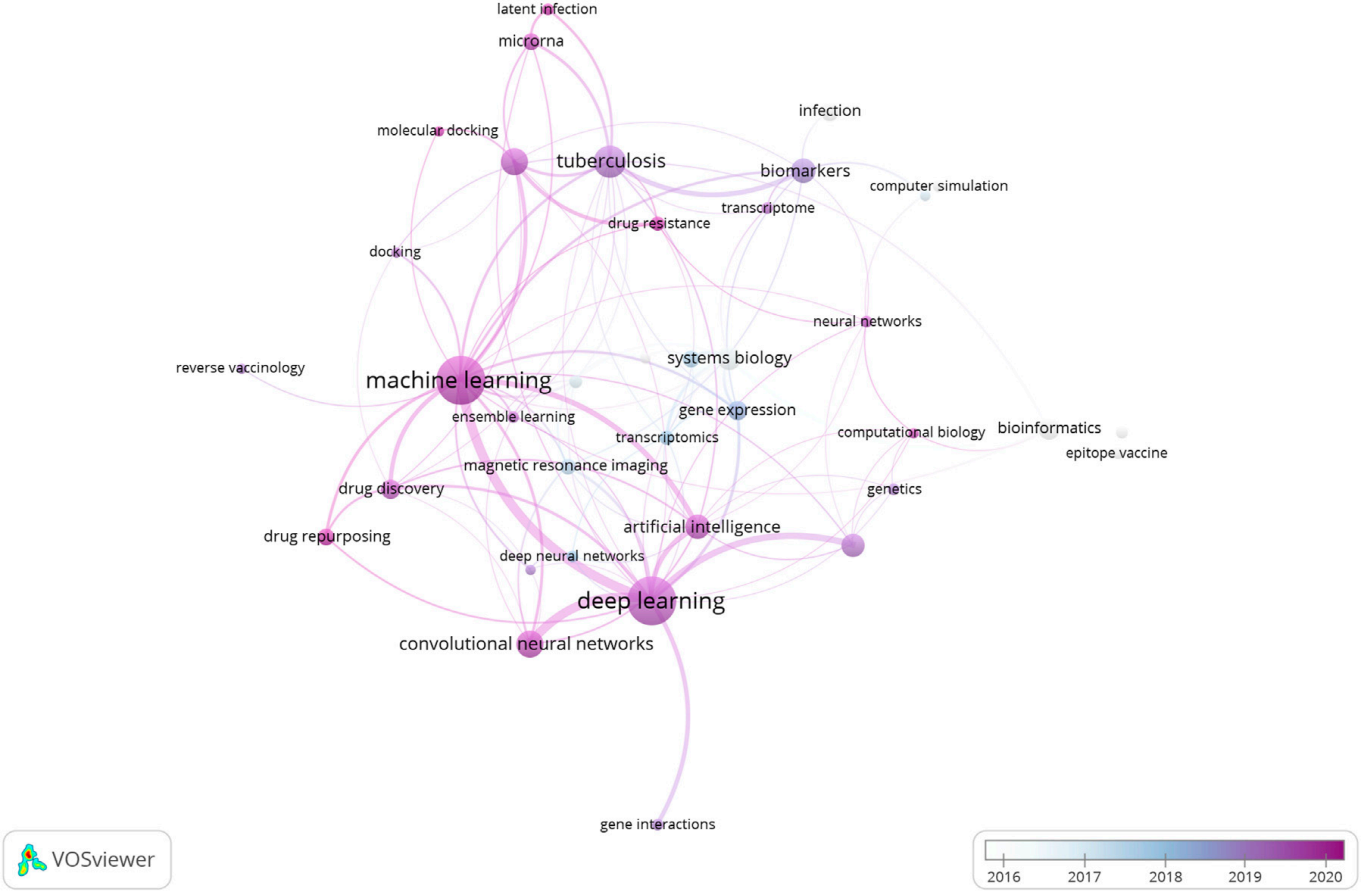
Network of representative keywords retrieved from the research papers included in the review article. The network clearly indicates the dominance of Machine Learning and Deep Learning algorithms in the research and development domain linked with Tuberculosis.

In the coming decade, as we leap towards the deadline set for the “END TB” strategy, the role of Machine Learning algorithms in every aspect of TB research cannot be underestimated. Figure 4 illustrates a network of the major “keywords” linked with the research articles included in the current literature survey. As it can be observed, the prominent presence of machine learning and deep learning algorithms in these papers is quite striking. It goes without saying that further standardization and optimization of these methodologies are expected based on requirements. For example, with regards to drug screening, building ML models to screen out active hit compounds (small/natural compounds) is a major challenge. The samples selected to train the model can heavily influence the precision and accuracy of it in determining the binding affinity to the target. Focusing on conformational interactions between drug and target, a group tried to neatly incorporate minority classes of drug conformations using an oversampling technique called synthetic minority oversampling technique (SMOTE), which based on regression, generate and add up more data which represent the minority class of conformation in the training datasets (to avoid sample biasness problem). This oversampling technique when combined with linear regression and k-nearest neighbour algorithm gave highest accuracy in predicting suitable target protein conformation (for ligand binding) as was validated experimentally (Akondi et al., 2022). A group successfully incorporated this technique to develop several ML models using a TB database to expand on the sample size. Minority (less sample size) sub-samples of interest of potential drugs with or without anti-tubercular properties can play an important role in the development of robust and sensitive ML models (Wani & Roy, 2022). Further elaborate studies are required for the identification, expansion and incorporation of these classes in the drug discovery process against TB.

Secondly, Deep Neural Networks (DNNs) can find vital applications in the fight against TB as their usage have been strongly advocated for biomedical use. The primary reason for why DNNs are so attractive is because—1) they offer a way to make sense of the huge amount of biochemical and molecular data currently being produced (which could aid in the drug discovery process), 2) there are parallels between the architecture of deep learning models and the conceptual framework of the central dogma which make the use of DNN models even more tempting to unravel the pathogenicity of the disease and to decode how the immune system responds to the host (Mamoshina et al., 2016). For example, Pairwise input Neural Networks (PNN)could provide a fantastic opportunity to simultaneously characterize and rank many drug-ligand interactions for a faster screening process. Given that Mtb is a complex pathogen and that the need of new drugs have become more and more urgent with the advent of drug resistance, PNN provide a strategic pipeline to reduce the time of discovery and to come up with credible drug candidates. Based on the similar concept, DeepBind algorithm provide information on the ability of candidate transcription factor and RNA-binding proteins to bind to a given genetic sequence. This tool can provide information on the pathogenesis of Mtb, the immune evasion and the resultant hyper (immune) reactivity—which might open doors for therapeutic interventions, including non-coding RNA (Mamoshina et al., 2016). Other tools which can be used to predict RNA-protein interaction are summarized in a review article by Zhong et al. (2021) which presents IPMiner and RPIScan, tools (both of which uses deep learning autoencoder networks along with Random Forests classifiers) as promising tools to predict RNA-protein interactions. These tools can be immensely valuable in creaming out credible non-coding RNAs as therapeutic agents (in conducting drug-protein interaction studies) and also in understanding RNA-protein interactions during TB pathogenesis for identifying key diagnostic motifs.

For better evaluation of ligand libraries used for FBDD, integration of **QSAR** based multi-scale models (which are able to integrate biological and chemical features of a compound whose query structure have been provided) have been suggested. This approach needs to be explored further with urgency against TB to come up with highly credible group of targets and their respective inhibitory fragment/fragments for the development of a multi-target treatment option which would be instrumental as we increasingly get short of treatment options. Efforts in this direction have already begun (Speck-Planche et al., 2012). Also, many advanced computational applications are also now available for drug screening and to perform advanced analysis on binding studies and can be used to bring out highly credible anti-TB drug candidates for wet lab studies post *in silico* analysis (Kleandrova & Speck-Planche, 2020). An important example is inverse molecular docking which begins with a selected ligand and used to prioritize target proteins from a set of pre-determined molecular targets. CANDOCK, the inverse molecular docking programme have been used to screen out natural compounds against host targets involved in TB. In this study, binding sites were identified for small-molecule binding before preparing them for inverse docking. Post docking, target molecules can be ranked based upon their docking score—revealing the best target-drug pair which along with being instrumental in the drug discovery process, can also provide invaluable information on the key host pathways involved in TB pathogenesis and progression. (Jiménez-Luna et al., 2021).

Advanced simulations often generate enormous data sources containing a high proportion of MD trajectories and millions of molecular structures. Primary challenge that Molecular Dynamics faces is to make sense of this data and to extract meaningful information of the structural complex under consideration as quickly as possible (Shao et al., 2007). The process of conformational clustering groups geometrically similar MD conformations into a cluster and is a useful approach to addressing this challenge as it allows for the study of thermodynamic properties of millions of MD conformations in great depth by dividing them into different clusters based on the associate properties (Gyebi et al., 2021).Many clustering algorithms established in computer programming have already been effectively implemented for MD datasets. The most frequently used clustering algorithms can be split into two distinct categories based on their basic principles: hierarchical and partitional clustering algorithms. And its subtypes. It is important to note that there is no “one-size-fits-all” clustering algorithm and they can be used based on the research requirements considering their respective merits and limitations (Glavaški et al., 2022)(Nayak & Sundararajan, 2023). The **linear interaction energy (LIE)** analysis computes the non-bonded van der Waals (vdW) and electrostatic (ele) interactions for the compound with protein target and the compound in water to compare the bound and unbound state of the small molecule/ligand/compound as well as binding capacity of the compound. This analysis provides better approximations to experimental binding free energies to the results obtained after *in silico* analysis (Furlan & Bren, 2021; Nayak & Sundararajan, 2023). The advanced computational tools discussed above, highlight the fact that, in the age where machine learning based drug screening is increasingly used by researchers, the tools and concepts of structural biology still find relevance to characterize molecular level receptor-ligand interactions at an atomic level to churn out high quality drug candidates post *in silico* analysis.

Last but not the least*, in silico* evaluation of potential drug toxicity provide an extraordinary opportunity to bring up credible lead compounds. Efforts have already begun to co-aggregate data from *in vitro* and *in vivo* experiments from different studies to have a comprehensive database of chemical structures and their associated biochemical properties like ACToR (Aggregated Computational Toxicology Resource). Such databases would act as important resources to feed machine learning models aimed at predicting toxicity. Also, the advent of miRNA technologies for preventive and therapeutic interventions, have created a new dimension for toxicity studies wherein the implication of miRNA-target binding in Tuberculosis can exhaustively be evaluated to screen out the safest miRNA candidates for further optimization (Vo et al., 2020).

As discussed above, the other application of machine learning algorithm would be in the screening of drug resistant strains of Mtb based on the genomic data of the pathogen. Although attempts are being made to keep track of the variants locally and globally, it is essential that a standardised protocol is drafted and deployed for whole genome sequencing analysis and for prompt identification of drug resistant variants (Ley et al., 2019). This would greatly accelerate the process of variant detection and characterization while making the derived results more credible. A great starting point in this direction would be development of a well-calibrated (well trained) ensemble model (A. Zhang et al., 2021) which can include appropriate machine learning models to capture the mechanism behind antibiotic resistance and in the process act as a universal predictive toolbox - handy for the clinicians and diagnostic centres to rapidly screen out antibiotic resistant strains. Lastly, for vaccine development and pre-clinical/clinical evaluation, defining of co-relates of protection (CoP) can greatly facilitate immune-monitoring and hence accelerate the regulatory process post vaccine development. Machine learning algorithms are recently being used assign immune signatures of protection for other diseases. Given that protective immune response to TB is yet to be elucidated, systems biology tools along with ML provide great prospects of research in establishing the CoPs against TB (Arevalillo et al., 2017).

### Limitations of using computational tools in TB research

Primary limitation of the use of computational tools and in using heavy data in research and development sector is the cost associated with it. Hardware and software with high computing power have hefty pricing raising questions on affordability. On the other hand, use of ML algorithm to establish diagnostic biomarker would require considerable thought on the possible study biases (like, sampling bias, exclusion biases and measurement biases. Moreover, it is important to note that the quality of the findings from ML algorithms are dependent on high quality structured data which could often be difficult to obtain in medical settings baring to infrastructural limitations. In molecular simulation studies for drug discovery, the primary challenge remains to be the setting up of parametric values to be as close to the biological conditions which are pretty dynamic. Another major ethical concern in the use of *in silico* approaches is associated with the need of storage, privacy and security of obtained biological data—legal and practical guidelines for the same are yet to be drafted and deployed.

## Conclusion

The current review takes a comprehensive approach to discuss key monitoring and interventional strategies under development against TB using computational and systems biology approaches. Due to its broad approach the articles provide only a brief overview of several advanced computational tools, details on which can be obtained in the cited articles. In conclusion, the success of the “END TB” strategy is dependent on the collective focus of the scientific community in developing and employing advanced methodologies to screen, manage and prevent the spread of Mtb strains specially in the endemic settings. Computational tools provide an unprecedented opportunity to make use of the available data to—increase our understanding of the disease, screen out the best drug/vaccine candidates and to develop clinically usable tool-box for tracing and hence managing antibiotics resistance. Through this review, we try to provide the current status of development of these applications while presenting exciting future prospects of research and development in this field.
