# Supplementary material for: Advances in computational frameworks in the fight against TB: The way forward
Source: Front Pharmacol. 2023 Apr 3;14:1152915. doi: 10.3389/fphar.2023.1152915 (PMC10106641; doi:10.3389/fphar.2023.1152915)
Supplement: Supplementary file 1 [file Table1.DOCX]

Basic Concepts in Computer Aided Drug Design (CADD)

1. **Virtual screening (VS)** is a procedure to identify leads (drug candidate) across a large and extensive library of bioactive molecules. VS is an important technique for developing lead compounds because it is faster, less expensive, and requires fewer resources than an experimental approach such as high-throughput screening. VS computationally screens libraries of specific types of drug-like or lead-like bioactive components against well-known three - dimensional structures of molecular targets (Chandrashekharappa et al., 2019; Heo et al., 2022; Mi et al., 2022; Motamen & Quinn, 2020; Puhl et al., 2020; Si et al., 2022; Zhang et al., 2018). Virtual screening of natural compounds such as alkaloids, coumarins, flavonoids have become an important tool for therapeutic applications, particularly those aimed at anticancer drug discovery. It has received considerable attention because it facilitates users to develop alternative strategies by selecting the most suitable set of compounds, trying to minimize expenditures of resources and enhancing unwanted compounds (Chandrashekharappa et al., 2019). This technique has many advantages including offering better hit rate, potential to study a wide of natural compounds against an array of targets and prediction of ADMET properties.
2. The quantitative structure-activity relationship (**QSAR**) is an algorithmic or statistical design approach often used to uncover connections between bioactive molecules and chemical small molecules structural features (Kingdon & Alderwick, 2021). The main objective is that comparisons in mechanical characteristics result in different pharmacological properties. Physicochemical properties are referred to as structural properties, and bioactive compounds have defined pharmacokinetic functions such as absorption, distribution, metabolism, excretion, and toxicity (Unni P et al., 2020). QSAR modelling, as an *in-silico* methodology, aids in the prioritisation of a wide range of compounds based on their preferred biological properties, significantly reducing the number of shortlisted compounds that are tested in vivo. QSAR mathematical modelling has evolved into an essential procedure in the healthcare industry, despite the numerous constraints (Neves et al., 2018).  Machine-learning approaches are now being used to predict QSAR to increase the creadibility and efficiency of the derived ligand. For QSAR prediction, linear regression models and Bayesian neural networks were used. Random forest (RF) is the most widely used algorithm because of its high predictability, simplicity, and robustness (Huang et al., 2021). RF uses decision trees and hence refrain from overfitting which could be caused by a single decision tree. For this reason, recently, QSAR prediction methods are frequently compared to RF (Tsou et al., 2020).

The primary objective of quantitative structure activity relationship (QSAR) or quantitative structure property relationship (QSPR) is to find/ predict statistical associations between drug’s biological activity and its molecular descriptors (Zi et al., 2021). To identify geometric descriptor 3-D molecular models are fed as input (Mahalakshmi & Jahnavi, 2020) which is followed by extraction of molecular structure descriptors (Ambure et al., 2019). The third step is to characterize the most important features or the molecular descriptors being used. Then QSPR and QSAR models are developed using descriptor sets selected in the previous step. The model is then validated using the text set (Ambili et al., 2020). To easily understand the model's fitness level, compare the predictions' results to those obtained for the training and cross-validation sets (Cai et al., 2022). There are a few applications that have achieved excellent results using the QSAR method:

1. Evaluation of novel prodrugs with greater energy levels
2. Better understanding and exploration of both pharmaceutical and chemical mechanisms of action
3. Drug candidates are improved to produce less toxic metabolites
4. Wet laboratory experimentation rationale: QSAR offers an economical and time-effective alternative to the medium-throughput in vitro and low-throughput in vivo assays.
5. Expense, duration, as well as resource specifications, are limited by creating more efficient biomolecules and using a less biologically exhaustive strategy; and finally,
6. to create alternatives to animal testing in accordance with the Registration, Evaluation, and Authorization of Chemicals (REACH) regulations.

Different steps are involved in QSAR method (Neves et al., 2018), the following steps are:

1. ***Molecular preparation for the QSAR experiment:*** Acquire a multi-locus set of inhibitors that have been checked in an essential physiological assay and demonstrated a broad range of action.
2. ***Descriptor selection in the training set:*** Enumerate the molecular descriptors aligned with the pharmacological activities of the phytochemicals.
3. ***Determine Descriptor Values in the Training Set:*** At stochastic, divide the compounds into two categories: training and test sets. Define and measure the correlation matrix that can explain the connection among descriptor values and bioactivity using its training dataset.
4. ***Both internal and external validation evaluation*:** Evaluate the statistical equation's stability using the test set molecules. A statistical model can be used to predict the biological activity of a novel chemical.
5. **Molecular docking** is an in-silico technique for representing the binding position of biological systems or ligands within the protein surface of their target protein. Two or more molecules, including protein and protein or a ligand and protein, could also form a bond in a broad range of ways in docking studies. As a result, docking studies aid in the identification of interactions formed between protein-ligand complexes as well as the nature of the interaction responsible for the inhibitory activity of the molecule. The molecular docking method is built on the search algorithm and scoring functions for creating and analysing ligand conformations. The most popular molecular docking tools are Autodock, AutoDock Vina, CDOCKER, GLIDE, DOCK6, GOLD, FLEXX, and SwissDock. Molecular docking approaches for ligand and receptor flexibility include: (1) rigid docking, in which the ligand and target structures are both rigid; (2) semi-flexible docking, which is the most commonly used approach, in which the ligand structure is flexible but the target structure is rigid; and (3) flexible docking, in which the ligand and target structures are both flexible. Various search algorithms, such as strategic search algorithms, random or stochastic algorithms, and simulation algorithms, are used to deal with flexible ligands. The precision of molecular docking is determined by hyperparameters that are used to determine strong interactions as well as putative binding modes, in addition to identifying potential candidate molecules.
6. **Molecular Dynamic Simulation (MDS)** is a tool used to model the structure dynamics of the target-drug complex after their binding to determine complex stability and characterize the binding properties (associated energies and the H-bonds involved). Although, deeper understanding of biomolecules at the atomic level, availability of 3D- protein structure and bioinformatics tool like homology modelling have greatly benefited structure-aided drug design, MDS founds its application in creating a virtual environment representing physiological conditions to track the conformational changes of the complex. Small to major conformational changes are expected in target protein when it binds to the ligand in the physiological environment and tracking the trajectory of the conformational changes becomes vital to check on the stability and feasibility of the formed complex (Nayak & Sundararajan, 2023).

MDS firstly requires a 3D protein structure of the target protein and a solvent model – which can be either explicit or implicit. Use of explicit solvents is simple and it can retrieve native solvent effects of the protein structure(Prabitha et al., 2022). For larger systems, an implicit solved can be used to speed up conformational sampling by using approximate parameters to the system. But this might in the long run effect the free energy values. The third most important element of MDS is the force field which is basically used to define different forces which are felt by the individual atoms of the system to determine the potential energy of the protein-ligand/protein-protein complex(Mugumbate et al., 2021). Currently, CHARMM, Amber and GROMOS are used to simulate molecular systems. In these force fields, Coulomb’s law is employed to characterize the electrostatic and van der Waals interactions, periodic functions are used to represent molecular/bond rotation and Lennard-Jones potentials and springs which represent bond length and angles(Kingdon & Alderwick, 2021). Then during atomic movements, acceleration and velocity of individual atoms are computed using Newton’s law of motion. Lastly, optimization processes are conducted to adjust for steric hinderances and stabilize the temperature-pressure system. Ones the system is equilibrated, the production run can be conducted for a certain time-period based on research requirements(Prabitha et al., 2022). After the simulation, stability of the complex can be evaluated using root-mean-square deviation (RMDS), root-mean-square fluctuation (RMSF) and radius of gyration (gives information on the flexibility of the protein). Moreover, allosteric interaction can be evaluated by generation protein energy/structure networks(Djaout et al., 2016).

**References:**

Ambili, Unni. P., Pillai, G. G., & Sajitha, Lulu. S. (2020). Integrated Ligand and Structure based approaches towards developing novel Janus Kinase 2 inhibitors for the treatment of myeloproliferative neoplasms. *BioRxiv*, 2020.11.26.399907.

Ambure, P., Halder, A. K., González Díaz, H., & Cordeiro, M. N. D. S. (2019). QSAR-Co: An Open Source Software for Developing Robust Multitasking or Multitarget Classification-Based QSAR Models. *Journal of Chemical Information and Modeling*, *59*(6), 2538–2544. https://doi.org/10.1021/acs.jcim.9b00295

Cai, Z., Zafferani, M., Akande, O. M., & Hargrove, A. E. (2022). Quantitative Structure-Activity Relationship (QSAR) Study Predicts Small-Molecule Binding to RNA Structure. *Journal of Medicinal Chemistry*, *65*(10), 7262–7277. https://doi.org/10.1021/acs.jmedchem.2c00254

Chandrashekharappa, S., Venugopala, K. N., Venugopala, R., & Padmashali, B. (2019). Qualitative anti-tubercular activity of synthetic ethyl 7-acetyl- 2-substituted-3-(4-substituted benzoyl) indolizine-1-carboxylate analogues. *Journal of Applied Pharmaceutical Science*, *9*(2), 124–128. https://doi.org/10.7324/JAPS.2019.90217

Djaout, K., Singh, V., Boum, Y., Katawera, V., Becker, H. F., Bush, N. G., Hearnshaw, S. J., Pritchard, J. E., Bourbon, P., Madrid, P. B., Maxwell, A., Mizrahi, V., Myllykallio, H., & Ekins, S. (2016). Predictive modeling targets thymidylate synthase ThyX in Mycobacterium tuberculosis. *Scientific Reports*, *6*(December 2015), 1–11. https://doi.org/10.1038/srep27792

Heo, J., Koh, D., Woo, M., Kwon, D., de Almeida Falcão, V. C., Wood, C., Lee, H., Kim, K., Choi, I., Jang, J., Brodin, P., Shum, D., & Delorme, V. (2022). A combination screening to identify enhancers of para-aminosalicylic acid against Mycobacterium tuberculosis. *Scientific Reports*, *12*(1), 1–14. https://doi.org/10.1038/s41598-022-08209-w

Huang, T., Sun, G., Zhao, L., Zhang, N., Zhong, R., & Peng, Y. (2021). Quantitative structure‐activity relationship (QSAR) studies on the toxic effects of nitroaromatic compounds (NACs): A systematic review. *International Journal of Molecular Sciences*, *22*(16). https://doi.org/10.3390/ijms22168557

Kingdon, A. D. H., & Alderwick, L. J. (2021). Structure-based in silico approaches for drug discovery against Mycobacterium tuberculosis. *Computational and Structural Biotechnology Journal*, *19*, 3708–3719. https://doi.org/10.1016/j.csbj.2021.06.034

Mahalakshmi, P. S., & Jahnavi, Y. (2020). a Review on Qsar Studies. *International Journal of Advances in Pharmacy and Biotechnology*, *6*(2), 19–23. https://doi.org/10.38111/ijapb.20200602004

Mi, J., Gong, W., Wu, X., & Al Attar, A. M. (2022). Advances in Key Drug Target Identification and New Drug Development for Tuberculosis. *BioMed Research International*, *2022*. https://doi.org/10.1155/2022/5099312

Motamen, S., & Quinn, R. J. (2020). Analysis of Approaches to Anti-tuberculosis Compounds. *ACS Omega*, *5*(44), 28529–28540. https://doi.org/10.1021/acsomega.0c03177

Mugumbate, G., Nyathi, B., Zindoga, A., & Munyuki, G. (2021). Application of Computational Methods in Understanding Mutations in Mycobacterium tuberculosis Drug Resistance. *Frontiers in Molecular Biosciences*, *8*(September), 1–11. https://doi.org/10.3389/fmolb.2021.643849

Nayak, S. S., & Sundararajan, V. (2023). Robust anti-inflammatory activity of genistein against neutrophil elastase: a microsecond molecular dynamics simulation study. *Https://Doi.Org/10.1080/07391102.2023.2170919*, 1–17. https://doi.org/10.1080/07391102.2023.2170919

Neves, B. J., Braga, R. C., Melo-Filho, C. C., Moreira-Filho, J. T., Muratov, E. N., & Andrade, C. H. (2018). QSAR-based virtual screening: Advances and applications in drug discovery. *Frontiers in Pharmacology*, *9*(NOV), 1–7. https://doi.org/10.3389/fphar.2018.01275

Prabitha, P., Shanmugarajan, D., Kumar, T. D. A., & Kumar, B. R. P. (2022). Multi-conformational frame from molecular dynamics as a structure-based pharmacophore model for mapping, screening and identifying ligands against PPAR-γ: a new protocol to develop promising candidates. *Journal of Biomolecular Structure and Dynamics*, *40*(6), 2663–2673. https://doi.org/10.1080/07391102.2020.1841677

Puhl, A. C., Lane, T. R., Vignaux, P. A., Zorn, K. M., Capodagli, G. C., Neiditch, M. B., Freundlich, J. S., & Ekins, S. (2020). Computational approaches to identify molecules binding to mycobacterium tuberculosis KasA. *ACS Omega*, *5*(46), 29935–29942. https://doi.org/10.1021/acsomega.0c04271

Si, A., Landgraf, A. D., Geden, S., Sucheck, S. J., & Rohde, K. H. (2022). Synthesis and Evaluation of Marine Natural Product-Inspired Meroterpenoids with Selective Activity toward Dormant Mycobacterium tuberculosis. *ACS Omega*, *7*(27), 23487–23496. https://doi.org/10.1021/acsomega.2c01887

Tsou, L. K., Yeh, S. H., Ueng, S. H., Chang, C. P., & Song, J. S. (2020). *Comparative study between deep learning and QSAR classifications for TNBC inhibitors and novel GPCR agonist discovery*. 1–11. https://doi.org/10.1038/s41598-020-73681-1

Unni P, A., Sudhakaran, S. L., & Pillai, G. G. (2020). Review on druggable targets of key age-associated properties regulated by therapeutic agents. *Chemical Biology and Drug Design*, *96*(4), 1069–1083. https://doi.org/10.1111/cbdd.13759

Zhang, M., Prior, A. M., Maddox, M. M., Shen, W. J., Hevener, K. E., Bruhn, D. F., Lee, R. B., Singh, A. P., Reinicke, J., Simmons, C. J., Hurdle, J. G., Lee, R. E., & Sun, D. (2018). Pharmacophore Modeling, Synthesis, and Antibacterial Evaluation of Chalcones and Derivatives. *ACS Omega*, *3*(12), 18343–18360. https://doi.org/10.1021/acsomega.8b03174

Zi, A., Laitinen, T., Patel, J. Z., & Poso, A. (2021). *Derivatives as FAAH Inhibitors*.
